# Cell cycle proteins: Linking the cell cycle to tumors

**DOI:** 10.32604/or.2025.058760

**Published:** 2025-05-29

**Authors:** JIE ZHONG, JUE LIU, XING TANG, WENCHAO ZHOU, GUANGMING SONG, YUHUAN ZENG, XIAODI ZHANG, JIANBIN ZHOU, JieZhou, LU CAO, QUNFENG ZHANG, YUKUN LI

**Affiliations:** 1 Department of Obstetrics and Gynecology, The Second Affiliated Hospital, Hengyang Medical School, University of South China, Hengyang, 421001, China; 2 Department of Assisted Reproductive Centre, Zhuzhou Hospital Affiliated to Xiangya School of Medicine, Central South University, Zhuzhou, 412000, China

**Keywords:** Cell cycle, Cyclin, Cyclin dependent kinase, Cell cycle checkpoint, Tumors

## Abstract

The cell cycle is a tightly coupled series of events that enable cells to grow and proliferate. Cyclin-dependent kinases (CDKs) play crucial roles in the cell cycle by enabling cells to transition between different phases when they are activated. Cell cycle proteins enhance the activity of CDKs, while natural CDK inhibitors (CDKIs) suppress them. The cell cycle continues in cycles under normal conditions, but when conditions change, cells halt or terminate the cell cycle. Tumors are tissues that grow out of control, and the mechanisms of various types of tumors are different; however, almost all tumor cells share several common characteristics, including proliferation, prevention of apoptosis and genomic instability. Cellular division is essential in the progression of cancer. A key characteristic of cancer is the uncontrolled growth of tumor cells, which is due to the erratic behavior of several proteins during the cell cycle. Therefore, cell cycle regulators are considered attractive targets for the treatment of cancer. The present analysis highlights proteins that play a direct role in controlling the tumor cell cycle, such as CDKs, and provides a brief overview of checkpoint kinases. The present review also discusses how cell cycle proteins contribute to cancer and describes some of the antitumor drugs that are being researched.

## Introduction

The cell cycle is the entire process of a cell from growth and replication to division. The cell cycle consists of two main phases, namely, mitosis and interphase. The M phase corresponds to mitosis, whereas interphase is subdivided into the G1, S, and G2 stages [1]. Among them, the cell division phase (M phase) and the DNA replication and synthesis phase (S phase) are the key phases in the cell cycle. Conversely, the G1 and G2 stages are when the necessary resources and conditions for DNA replication and cell division are prepared. Under normal circumstances, the cell carries out these four phases in a certain order and maintains a continuous cycle for normal cell growth and division. The regularity of the whole process is determined by a complex set of regulatory systems, and the precise replication and segregation of DNA is the ultimate purpose of the complex regulatory systems that make up the cell cycle [2]. In the cell cycle, events occur in a temporal sequence, with precision and parity. However, this process can go wrong and lead to cellular carcinogenesis and tumor growth if DNA is damaged or if cell division is uncontrolled. Tumor cells usually have a defective DNA damage checkpoint that allows them to continue dividing despite their imperfect cell cycle [3]. During the tumor cell cycle, certain important regulators lose the balance they maintain between them, such as proto-oncogenes and oncogenes, resulting in unlimited proliferation of tumor cells. In addition, abnormalities in cell cycle proteins are common in human tumors [4]. Therefore, the study of the cell cycle regulatory system can help identify more accurate treatment options for tumors.

The present review highlights key proteins, cell cycle checkpoints and their relevance to cancer and development, and it provides a concise summary of the cell cycle regulatory pathway. However, a complete description of the entire process of the cell cycle is impossible because of the sheer size and detail of the process. Therefore, the present review focuses on several processes and briefly mention others.

## Cell Cycle Regulation

**Figure 1:**
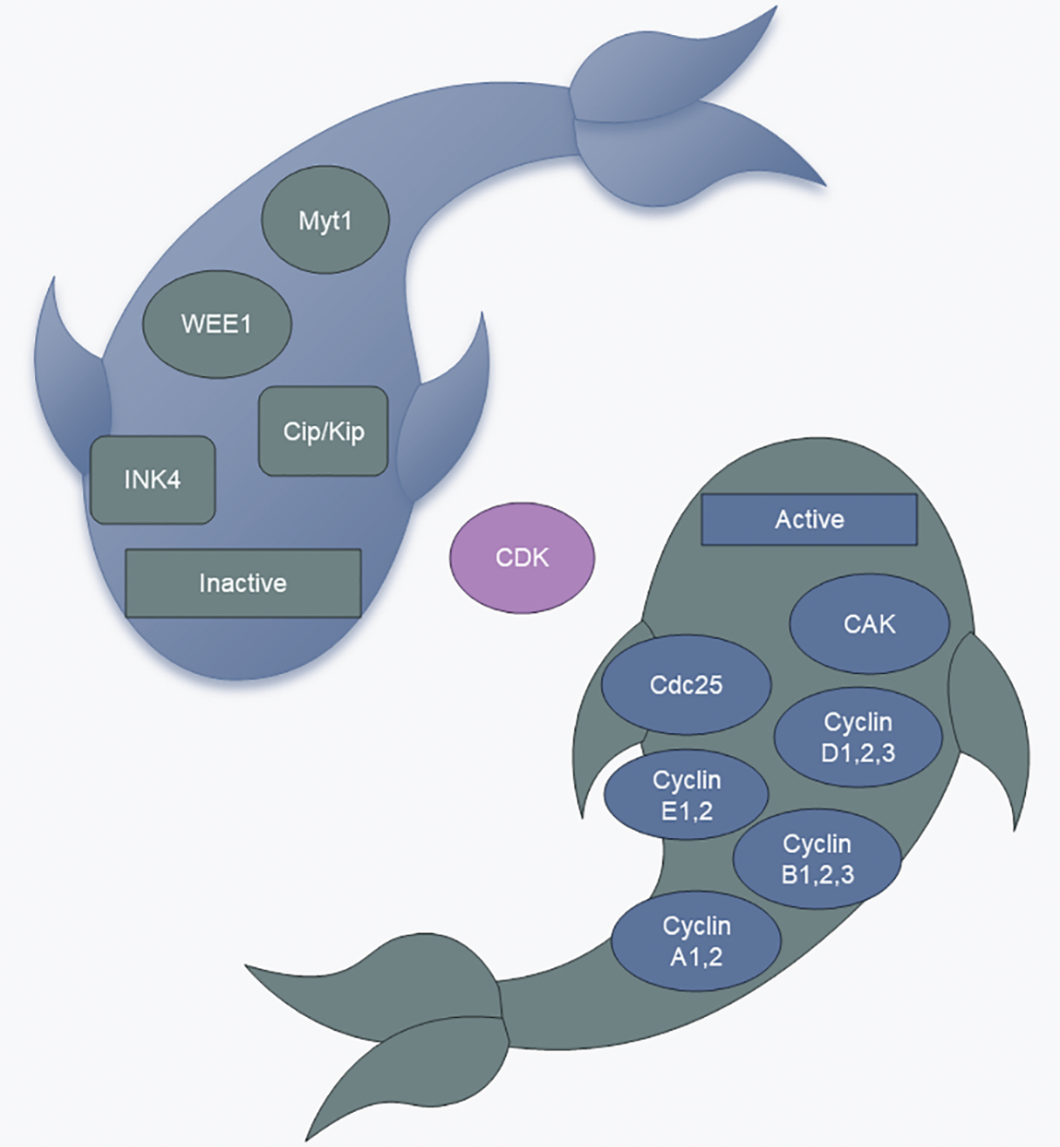
CDK regulators. CAK: CDK-activated kinase, Cdc25: cell division cyclin 25 (Fig. 1 uses templates from Microsoft PowerPoint).

Cell cycle regulation relies on a complex network of cell cycle regulatory proteins. The cell cycle regulatory system has fine molecular switches that allow specific biochemical reactions to be activated and inactivated at defined times. Proteins involved in the cell cycle and kinases that rely on these proteins are crucial to the overall regulatory framework [2]. In mammals, cyclins encompass cyclin A through H and cyclin T. Cyclin-dependent kinases (CDKs) are protein kinases that require binding to cell cycle proteins to become active, and they are essential for controlling the cell cycle by phosphorylating various proteins associated with the cell cycle. The CDK family members are categorized into those that play a direct role in the cell cycle and those that actively participate in the cell cycle. The cell cycle process involves CDKs, such as CDK1, CDK4, CDK5, and CDK6, as well as transcriptional regulators, such as CDK7, CDK8, CDK9, CDK11, CDK19, and CDK20; in addition, CDK7, CDK11, and CDK20 also play roles in cell cycle regulation [5]. Moreover, certain proteases, including cell division cyclin 25(Cdc25) phosphatase and CDK-activated kinase (CAK, CDK7/cyclin H), enhance CDK activity into control the cell cycle [6]. Conversely, CDK kinase inhibitors (CKIs) are also involved in regulating the cell cycle. Cells produce CKIs that inhibit the cell cycle during the G1 phase, thereby stopping the initiation of defective DNA replication and cell division. Mammalian CKIs are classified into the KIP/CIP (p21, p27, and p57) and INK4 (p16INK4A, p15INKB, p18INK4C, and p19INK4D) families, in which the KIP/CIP family maintains the stability of the cyclin D-CDK4/6 complex, inhibiting the activity of CDK1 and CDK2 [7]. Additionally, Aurora, Polo, WEE1, and Myt1 also play roles in cell cycle control [8] (Fig. 1).

**Figure 2:**
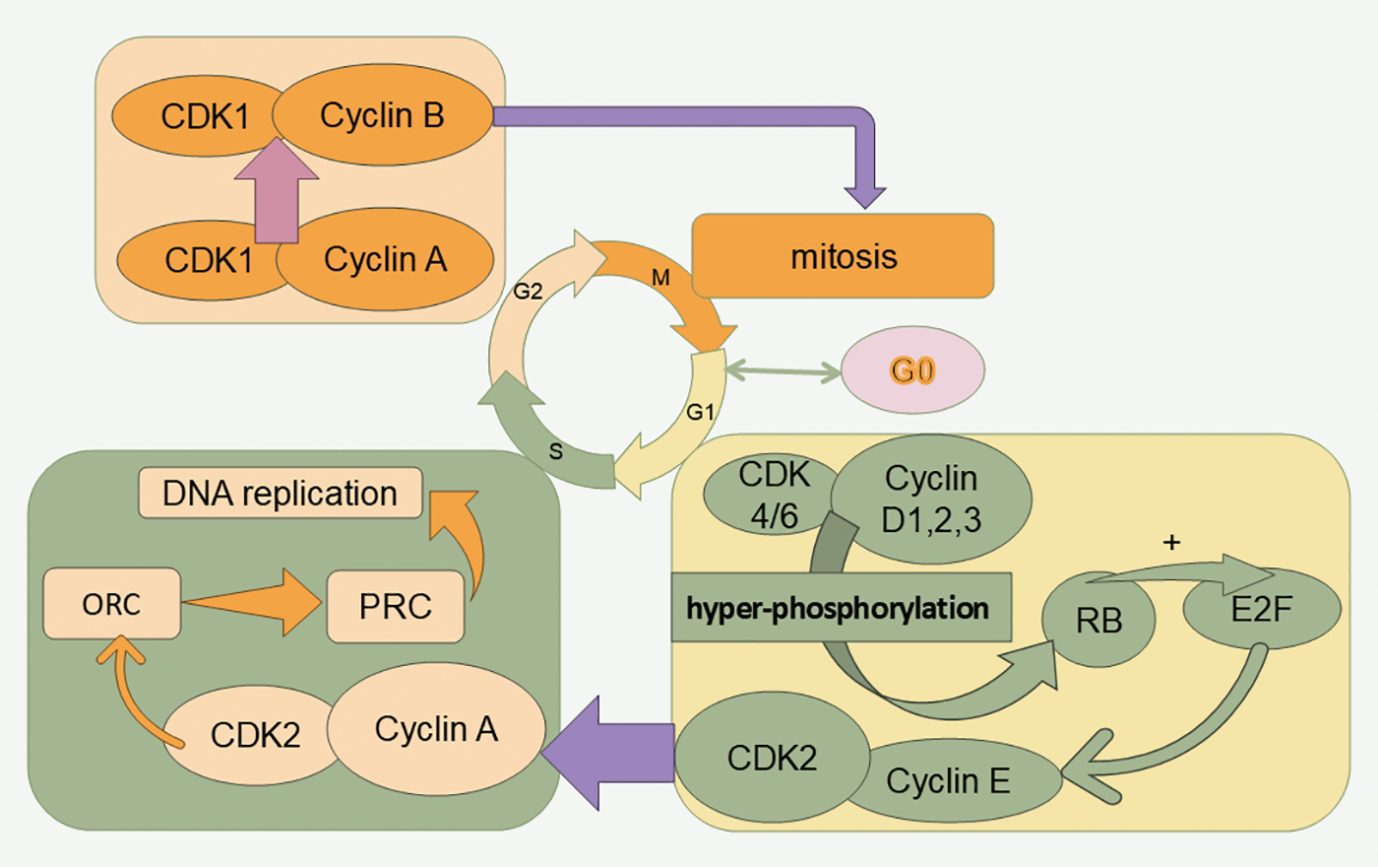
Cell cycle and related proteins. The cell cycle is divided into four phases (G1, S, G2, and M). Cell cycle progression is promoted by CDKs. RB: retinoblastoma protein, ORC: origin recognition complex, PRC: prereplication complex.

The currently recognized initiating event for cell cycle initiation is an extracellular stimulus to G0 cells that induces an increase in the transcription of cyclins (D1, D2, and D3), which then bind specifically to CDK4 or CDK6. Cyclin D-CDK4/6 complexes contribute to the expression of the E2F master cell cycle transcription factor through the hyperphosphorylation of the retinoblastoma (Rb) protein. Phosphorylation contributes to the expression of the E2F master cell cycle transcription factor [8–10]. When RB1 is inhibited, E2F activity is restored. Next, the binding of CDK2 to cyclin E, which is progressively more abundant in late G1, promotes RB1 phosphorylation, which in turn promotes downstream E2F transcriptional activity, prompting the cell to cross the restriction point (R-point) and allowing the G1 phase to transition into S phase [11]. Subsequently, cyclin A replaces cyclin E and binds to CDK2 and further stimulates DNA replication by phosphorylating cdc6. Cyclin A attaches to CDK1 and CDK2, and their binding promotes the production of cyclin B-CDK1, commonly known as maturation-promoting factor (MPF), which triggers the onset of the M phase [12,13]. The formation of the cyclin B-CDK1 complex facilitates chromosome condensation, breakdown of the nuclear envelope, spindle assembly, segregation of sister chromatids, and subsequent inactivation to ensure the completion of mitosis [14] (Fig. 2).

Under typical conditions, the cell cycle follows a G1-S-G2-M sequence. However, if there is an environmental shift or disruption in the cell cycle, the cell halts or ends the cycle to maintain proper progression [15]. This regulation is managed by cellular monitoring systems called checkpoints. The detection points include the unreplicated DNA detection point, the spindle assembly detection point, the chromosome segregation detection point, and the DNA damage detection point. The roles of each of these monitoring points will be discussed later. The genetic stability of cells can be maximized through a strict cell cycle detection system. In contrast, in cells that have lost the detection point mechanism, the genome is highly unstable, and the chances of DNA amplification, rearrangement, and point mutation increase, which can lead to tumorigenesis. In addition, during G1, cells can suspend the cell cycle and enter a quiescent state, also known as G0, a nonproliferative state. Several studies have shown that tumor cell rekindling and the G1/G0 transition are inextricably linked, which has been previously reviewed [16].

## Tumors and the Cell Cycle

Tumors are tissues with uncontrolled growth; the mechanism of different types of tumors varies greatly, but almost all tumor cells share several common features, including sustained proliferation, prevention of apoptosis and genomic instability. The occurrence of tumors is often inextricably linked to cell cycle disorders [17], including but not limited to the disorders discussed below.

Pathway abnormalities: The normal progression of the cell cycle relies on the regulation of a series of complex signaling pathways, and abnormal function or expression levels of regulatory factors may lead to malfunction of the cell cycle, which may in turn lead to tumors. Abnormalities in the detection points of the cell cycle can also lead to tumor development [18].

DNA damage and repair: When the balance of DNA damage and repair is disrupted, abnormal cell proliferation and division may occur, thus promoting tumor development and progression.

Oncogenes and proto-oncogenes: Most oncogenes and proto-oncogenes are components of signal transduction pathways that control various cellular functions such as cell cycle entry and exit. Oncogenes are key genes responsible for inhibiting cell proliferation and division, and their abnormal function or inactivation may lead to cell cycle disruption and cancer development [19]. Proto-oncogenes, on the other hand, are important genes that promote cell proliferation and division, and their abnormal activation can also cause cell cycle imbalance and cancer development. For example, p53 research has been popular since its discovery decades ago. A previous review has summarized in detail the tumor-related functions of p53 [20]. Some studies have revealed the association of p16 with the development of some malignant tumors [21].

Growth factors: Growth factors are a class of peptides produced by cellular autocrine or paracrine production, and they activate various intracellular protein kinases and promote or inhibit the expression of proteins related to the cell cycle process, including those involved in the regulation of the cell cycle. For example, transforming growth factor-beta (TGF-β) inhibits cell division during the cell cycle in most cell types, whereas it promotes cell division in a few cells of mesenchymal origin, such as osteoblasts. TGF-β induces tumors mainly through partial activation of epithelial‒mesenchymal transition (EMT), to induce tumor growth [22].

Previous studies have shown that tumorigenesis is frequently dependent on mutations in individual cell cycle proteins and CDKs [7]. Erroneous operation of the cell cycle regulatory system is a prerequisite for abnormal cell division. Errors in the segregation mechanism during cell division lead to chromosomal abnormalities, which produce progeny cells with abnormal chromosome numbers and contents. During cell division, there is an increased tendency for the genome to acquire mutations, which is defined as genomic instability. Genomic instability in tumor cells is thought to be one of the causes of cancer development, allowing tumor cells to proliferate in large numbers [23]. Mitotic catastrophe is a delayed death mechanism associated with cellular mitosis, and is an important mechanism for cancer suppression under physiological conditions. If mitotic catastrophe is not generated properly, the defective cells will grow unrestrictedly, which is a major pathway for tumor development. In recent years, studies have been conducted to treat tumors by activating this mechanism, and the main target is the cell cycle checkpoint [24]. Apoptosis is a mechanism by which a normal organism responds to abnormal cell production, but apoptosis can occur in tumor cells. Most tumors achieve infinite replication by evading apoptosis. Ferroptosis, which is a natural tumor suppressor mechanism, is currently a hot topic of research. Many tumor suppressors, such as p53, exert their tumor suppressor function through this ferroptosis. Drug-induced stimulation of ferroptosis is also a current direction in cancer treatment [25]. Cellular autophagy is likewise a mechanism by which the body disposes of abnormal cells, and it is primarily responsible for the degradation of damaged organelles and toxic macromolecules in the cell. The regulation of cellular autophagy cannot be separated from the regulation of cell cycle proteins, and they are usually aberrant in the regulation of the tumor cell cycle, which promotes or reduces cancer progression [26]. Mitotic slippage, the exit of cells from mitosis without proper chromosome segregation, is a typical response of TP53 mutant tumors resistant to genotoxic treatments. However, less research has been conducted on this mechanism, which may serve as a new target for cancer treatment [27]. Cellular senescence is a state of stable cell cycle arrest, and cells at this stage share many similar characteristics with tumor cells. Eradicating tumors by inducing a senescence response in cancer cells is also a hot topic at the present [28].

Thus far, the present review has discussed many mechanisms of tumorigenesis with possible therapeutic targets, such as cell fate after abnormal cell division, mitotic catastrophe, apoptosis, autophagy regulation, mitotic sliding, and cellular senescence, all of which are closely related to cell cycle regulation. Some of the tumor therapeutic agents related to cell cycle proteins are listed in Table 1. Because there are many mechanisms of tumorigenesis, it is impossible to completely summarize all the relevant elements, Thus, the present review focuses on the relationships of cell cycle proteins and cell cycle checkpoints with tumors.

**Table 1:** Oncology drugs related to cell cycle kinase inhibitors

Drug	Stage	Status	Cell cycle target	Tumor type	Reference
Dinaciclib (MK7965)	1/2	Completed	CDK1/2/5/9	Breast cancer	[113]
Ebvaciclib (PF-06873600)	1/2	Active, not recruiting	CDK2/4/6	Breast cancer	[114]
Ro-3306	Preclinical	Terminated	CDK1	Ovarian cancer	[115]
Abemaciclib	2	Active	CDK4/6	Glioblastoma	[116]
PF-07104091	2	Recruiting	CDK2	Breast cancer and solid tumors	[117]
Berzosertib + gemcitabine	2	Active	ATR	Ovarian cancer	[118]
GDC-0575	1	Active	CHK1	Solid tumors	[119]
AZD0156	Preclinical	Active	ATM	Glioblastoma	[120]
MK-1775	1/2	Completed	WEE1	Adenocarcinoma of the pancreas	[121]
Rigosertib	3	Completed	PLK1	Myelodysplastic syndromes	[122]
Alisertib	2	Completed	AURORA A	Acute myeloid leukemia	[123]

## G1 Phase: Preparation of DNA for Replication

The primary function of the G1 phase is to prepare for DNA replication and synthesis. During the G1 phase, protein synthesis and metabolic activities are vigorous, and the cell volume increases rapidly, along with extremely active material metabolism, mainly the synthesis of large amounts of RNA and proteins, the phosphorylation of many proteins, and the uptake of nutrients. In the late G1 phase, there is a critical point that determines whether the cell divides in the future, which is known as the R-point in mammalian cells, and a cell that has crossed the R-point no longer needs growth factor stimulation to divide [29]. The binding of cyclinE-CDK2 and cyclinD-CDK4/6 occurs in the late G1 phase, allowing the cell to cross the R point and transition to the S phase. During the normal cell cycle, growth factor stimulation increases cyclin expression, causing cyclin D- CDK4/6 activity to exceed a threshold. CyclinD-CDK4/6 adds phosphate groups to the Rb transcriptional inhibitor, partially increasing its ability to suppress the E2F transcription factor family. This activation increases the expression of numerous growth-related genes, such as cyclin E, thereby increasing cyclin E-CDK2 activity, and further phosphorylation creates a positive feedback loop through Rb hyperphosphorylation, permitting full activation of E2F and initiation of the cell cycle cycle [8].

CDK4/6 inhibitors (CDK4/6is) have been a relatively hot research topic in recent years [30]. Some drugs are now being used in the clinic after a long period of research. Palbociclib, ribociclib and abemaciclib are targeted drugs for the treatment of breast cancer. Their mechanism of action is mainly by blocking the binding of CDK4 and CDK6 to CDC37, the kinase-targeting subunit of HSP90, thus preventing CDK4/6 from entering the HSP90 chaperone system. In addition, CDK4/6 inhibitors inhibit tumor growth through several mechanisms such as promoting tumor cell senescence and altering the immune environment of tumor cells [31–33]. CDK4 plays a role in many stages of tumors. A previous review has summarized in detail the role of CDK4 in tumor cells [1]. CDK6 also plays an important role in the development of tumor cells. The progression of hepatocellular carcinoma can be affected by the knockdown of the long noncoding RNA H19 (H19) in hepatocellular carcinoma cells, and the expression of H19 is associated with CDK6. However, drugs that affect this process alone have not been identified [34]. Another review has summarized the progression of CDK4/6 through immunomodulatory sites to treat tumors [35]. The CDK4/6-RB pathway is commonly mutated in tumor cells. CDK4 is amplified in half of glioblastomas and, deletion of RB1 occurs in many tumors [36]. CDK6 is activated in splenic marginal zone lymphomas [37]. Some studies have shown that knockout of CDK6 increases resistance to induced tumorigenesis in mice [38]. Other studies have revealed an association of cyclin D with breast carcinogenesis [39]. Conversely, other animal studies have shown that mice deficient in cyclin D1 are resistant to breast cancer caused by specific oncogenes [40,41]. Recently, a new regulator of cyclin D: PC4 has been identified, and it may serve as a potential therapeutic target for tumors [42]. These studies highlight the connection of cyclin D1 and CDK4/6 with cancer development. Furthermore, numerous studies have revealed that CDK4/6 inhibitors are effective not only in the G1 phase but also in suppressing tumor growth during the S and G2 phases [43]. This action may be linked to internal replication within the cell cycle [44]. Additionally, a recent study has indicated that tumorigenesis can be effectively managed by targeting the CDK7-mediated CDK4/RB pathway and that FERM domain-containing 8 disrupts the interaction of CDK7 with CDK4, thereby inhibiting the activation of CDK4 to affect tumor growth [45]. Although CDK7 is not directly involved in cell cycle regulation, increasing evidence shows that inhibiting CDK7 decreases tumorigenesis [46,47]. While CDK2 mutations are uncommon in tumors, CDK2 complexes are often overactivated. For example, E2F-driven transcription of cyclin E (CCNE) at the CCNE1 site is often increased in human cancers [48–50]. Because the recent advancements in CDK2 research are inadequate, the link between CDK2 activity and cancer development remains uncertain. In certain tumors, cancer cells can continue to grow despite the inhibition of CDK2 [51]. CDK2 may play different roles in different tumors.

## S Phase: Replication Period of DNA

S phase is also known as DNA replication and synthesis, and the main features of S phase cells are DNA replication, chromatin protein synthesis and the formation of new DNA to chromatin. After the S phase, the content and activity of enzymes required for DNA synthesis, such as DNA polymerase, DNA ligase, thymidine nucleoside kinase, and nucleotide reductase, increase. The cyclinA-CDK2 complex, which is the most dominant cyclin-CDK complex in the S phase, initiates DNA replication and prevents replication of replicated DNA from occurring again.

The cyclinA-CDK2 complex triggers DNA replication, whereas the origin recognition complex (ORC), a large assembly of various proteins, attaches to the replication origin and acts as a hub for numerous regulatory proteins. At the beginning of the G1 phase, Cdc6 attaches to the ORC at the replication origin, and a set of deconjugating enzymes known as MCM also bind, forming a large protein assembly called the pre-replication complex (PRC) [52]. The cyclinA-CDK2 complex then uses its kinase function to phosphorylate specific sites on the PRC, triggering its activation and the start of DNA synthesis. In addition, the cyclinA-CDK2 complex can also activate certain DNA deconjugating enzymes in the prereplication complex through phosphorylation, and initiate DNA replication by dissociating the DNA double-strand and promoting enzymes related to DNA synthesis, such as the replication polymerases pol ε and pol δ [53–55]. RNA polysaccharides (RNPs) can also be activated through the phosphorylation of certain DNA deconjugating enzymes in the prereplication complex through their kinase activity [56–58]. The activity of RNA pol II also affects tumor cell growth [59].

After the initiation of DNA replication, Cdc6 dissociates from the ORC in the presence of the cyclinA-CDK2 complex, causing the reassembly of the prereplication complex, a mechanism that ensures that DNA cannot replicate again at the original replication initiation site. This action continues into the G2 and M phases so that DNA cannot be replicated until late mitotic chromosomes are separated from each other.

As mentioned earlier, CDK2 is not considered active in tumors, but the cyclinA-CDK2 complex is frequently overexpressed in tumor cells [60–62]. ATR is present during all cell cycle stages, but its connection to tumors is established through mitotic DNA synthesis (MiDAS) in the S phase [63]. ATR activity is essential for all highly proliferative cells, including tumor cells. Inhibition of ATR limits tumor growth. This process may be related to the involvement of ATR in cell cycle replication stress. Berzosertib is the first ATR inhibitor to be evaluated in humans and experiments on berzosertib are currently ongoing [64]. KI-67 is clinically utilized for tumor staging and has been recently identified as having a role in DNA replication, although more research is needed to understand its importance in cancer therapy [65]. CDK9 is a central regulatory hub at several stages of the transcription cycle. CDK9 also plays a key role in the repair of DNA damage. Most of the CDK9 inhibitors currently in clinical studies have strong cytotoxic side effects. Certain studies have demonstrated that CDK9 inhibitors exhibit superior effectiveness in treating clear cell renal cell carcinoma and breast cancer. XPW1 is a new CDK9 inhibitor that has recently been studied. XPW1 inhibits the aberrant transcription and DNA repair programs induced by CDK9, thereby blocking cell proliferation [66,67].

## Centrosome: The Key to Chromosome Segregation

Centrosome replication takes place during the late G1 and early G2 phases, whereas centrosome dynamics occur in the late G2 and early M phases. The key aspect of acentrosomal organization is the acentrosomal behavior of the centrosome [68]. Initially, a pair of centrioles positioned at right angles to each other separates, and each then generates a subcentriole in a perpendicular orientation. These newly formed centriole pairs act as microtubule organizing centers, contributing to the formation of spindle microtubules, astral microtubules, and more as the cell cycle process continues. Centrioles are essential for the G1/S phase, cell division, migration, polarity, and formation and orientation of the mitotic spindle [69].

Amplification of centrosomes is common in tumor cells [70], and inhibitors of centrosome amplification are effective in controlling tumorigenesis [71]. CDK1, CDK2 and CDK4, as well as cyclin A and cyclin E, have specific sites on centrosomes and play important roles in centrosome replication [72–76]. However, a recent study has proposed that the binding of CDKs to the centrosome does not strongly affect the overall process of DNA replication [77]. The question of whether CDK overexpression caused by amplified centrosomes is linked to tumorigenesis requires further investigation.

## G2 Phase: Preparatory Period for Cell Division

The G2 phase mainly prepares the cell for the division phase, during which the main proteins synthesized are those related to the structure and function of the M phase. Cyclin A and cyclin B interact with CDK1.The cyclin B-CDK1 complex, also known as MPF, primarily facilitates the shift from the G2 phase to the M phase.

CDK1 activity is rarely deregulated in cancer and is required for tumor formation. The inhibition of CDK1 prevents tumor development [78,79]. However, CDK1 is also required for the growth of normal tissues, so whether targeting CDK1 inhibition can be used to treat tumors clinically needs to be further explored. PLK1, which acts mainly in the G2 phase, controls the phosphorylation of CDK1 at the Tyr15 site via CDC25C or WEE1 [80]. Like most cell cycle-associated proteins, PLK1 is differentially expressed in tumor cells. Mutations in the core promoter region of telomerase reverse transcriptase (TERT) are relatively common. PLK1 interferes with TERT formation and inhibits the growth of hepatocellular carcinoma cells [81]. PLK1 is variably expressed in cancer cells. In general, PLK1 is viewed as an oncogene and a possible target for cancer therapies. PLK1 inhibitors have been shown in several studies to achieve promising results in the treatment of hepatocellular carcinoma [82].

## M Phase: Periods of Cell Division

M phase, also known as cytokinesis, is a period in which cells divide to equally distribute chromosomal genetic material to two daughter cells. Cells in the M phase have abundant and significant morphological changes and features as follows: separation of sister chromatids after chromosome condensation and disintegration; reconstruction of the nuclear membrane, formation and disappearance of the spindle, and appearance of contractile loops and cytoplasmic splitting. The start of mitosis is controlled by the activation of CDK1 and the cyclin B complex. When CDK1 activity surpasses a certain level, it initiates mitosis by phosphorylating numerous CDK1 targets, such as PLK1, Aurora A (AURKA), and Aurora B (AURKB) [56].

AURKA is considered an oncogenic kinase and mRNA mistranslation underlies its contribution to tumorigenesis [58] AURKB has also recently been found to be associated with tumorigenesis [57]. Tumor cells exhibit have chromosomal abnormalities, including chromosome number abnormalities and structural abnormalities [83]. Aneuploidy or polyploidy, which occurs due to the uneven distribution of chromosomes, is considered as one of the mechanisms driving tumorigenesis by gaining or losing chromosomes for relevant genes [84,85]. During cell division, the likelihood of genetic mutations, including chromosomal rearrangements, gains or losses, as well as nucleic acid sequence changes, increases [86]. To counteract the cytogenetic effects of this instability, several mechanisms have emerged. For example, the TIF 1 family (TRIM24, TRIM28, TRIM33, and TRIM66) is associated with DNA repair mechanisms [85]. Low-level expression of one of these proteins, TRIM33, is strongly associated with the development of certain cancers [87].

## Unreplicated DNA Checkpoints

During the normal cell cycle, cells cannot enter mitosis without DNA replication. The roles of unreplicated DNA checkpoints include recognition of unreplicated DNA and inhibition of MPF activation [88]. Two protein kinases, ATR and Chk1, have important functions at the unreplicated DNA checkpoint, preventing cells from dividing without DNA replication. In the process of DNA replication, ATR becomes active upon attaching to replication forks, triggering a chain of protein kinase reactions. ATR then phosphorylates and activates Chk1 kinase, which subsequently phosphorylates the Cdc25 phosphatase [89]. This prevents Cdc25 from removing the inhibitory phosphate group on M phase CDKs, keeping the cyclin B-CDK1 complex inactive and unable to phosphorylate proteins necessary for initiating M phase. The cyclin B-CDK1 complex remains inhibited and is unable to phosphorylate M phase target proteins. This activity continues until DNA synthesis is completed at all replication forks and the forks are disassembled, thus making it necessary for the M phase to occur only after the end of DNA synthesis. The relationship between these two protein kinases and tumors is described later.

## Spindle Assembly Checkpoint

**Figure 3:**
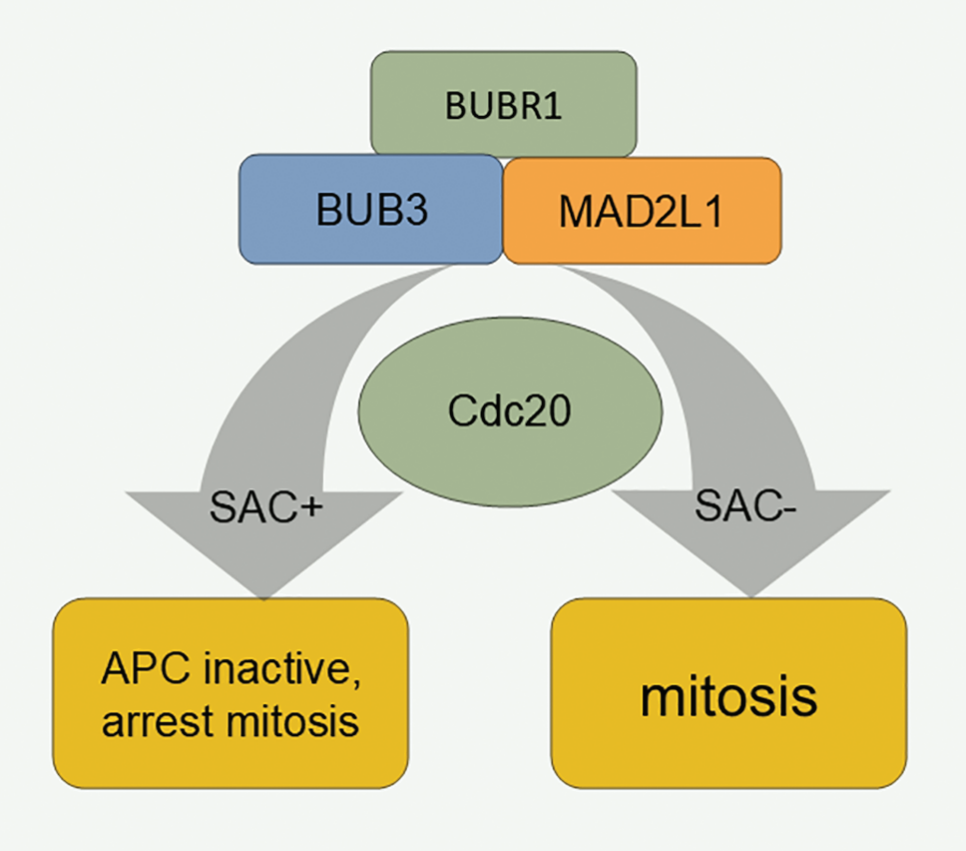
Mechanism of the spindle assembly checkpoint. SAC: Spindle assembly checkpoint.

The main function of the spindle assembly checkpoint is to stop cells with defective or unfinished spindle assembly from progressing to the later phase [90]. The spindle assembly checkpoint consists of BUB1, BUB3, BUBR1, MAD1L1, and MAD2L1. BUB1 plays a central role in the silencing and activation of the SAC. Studies of yeast checkpoint mutants have confirmed that MAD2L1 is the protein responsible for stability in the mechanism of action of the spindle assembly checkpoint [91]. Studies on yeast spindle assembly checkpoint mutants have confirmed that MAD2L1 is the protein responsible for the stability of the spindle assembly checkpoint mechanism of action [92]. During the cell cycle, APC-mediated polyubiquitination of sequin proteins controls the transition from the middle to late stage, and Mad2 inhibits Cdc20, an activator of APCs [93] (Fig. 3).

## Chromosome Segregation Checkpoint

During cell cycle progression, various events that occur at prophase and subsequent cytoplasmic divisions require the inactivation of MPF, and the activation of the Cdc14 phosphatase promotes the degradation of M phase cyclin via the polyubiquitination pathway, resulting in the loss of MPF activity and triggering the transition to prophase [94]. The chromosome segregation checkpoint is used to determine whether activated Cdcl4 phosphatase is produced in the cell by monitoring the position of segregating zygotic chromosomes in the post-end-of-phase cell, to promote the entry of the cell into the end-phase, cytoplasmic divisions, and, ultimately, exit from the M phase [95]. The presence of this test site prevents the onset of anaphase and cytoplasmic division before the zygotic chromosomes are correctly segregated, ensuring that the zygotic cells contain a complete set of chromosomes.

## DNA Damage Checkpoint

**Figure 4:**
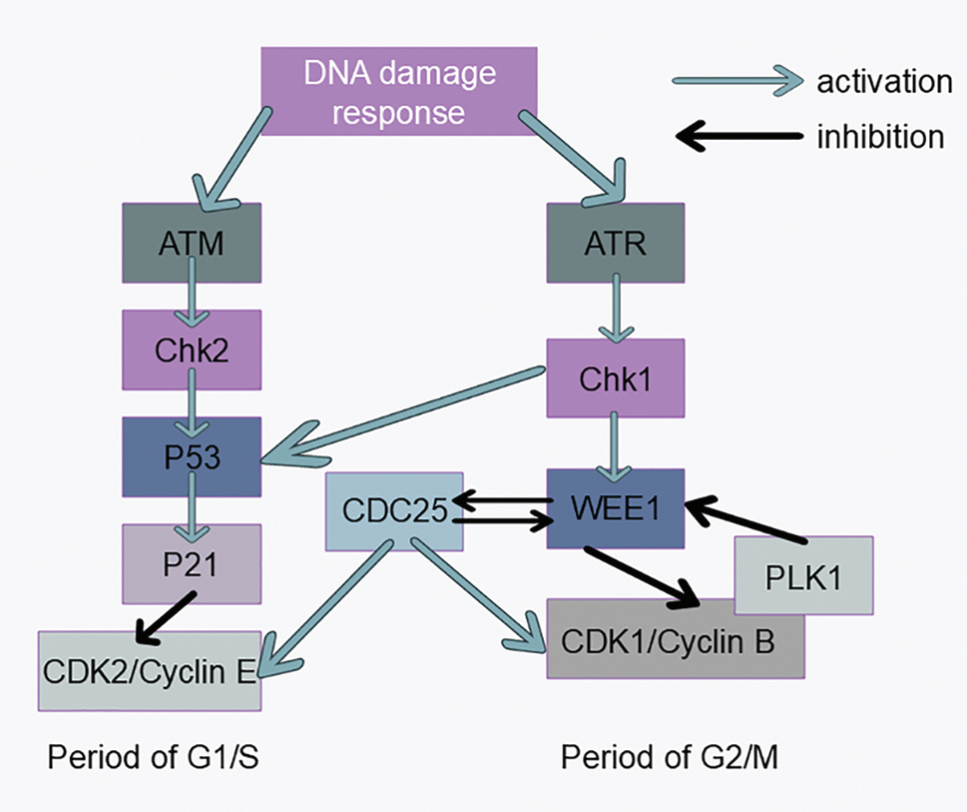
DNA damage response signaling pathways. Activation of the ATM-CHK2-P53 signaling pathway inhibits CDK2 expression, which affects progression from G1 to S phase. Activation of the ATR-CHK1-WEE1 signaling pathway inhibits CDK1 expression, which affects progression from G2 to M phase.

During the cell cycle, DNA may be damaged by external chemical and physical factors, and the DNA damage checkpoint prevents the cell cycle from continuing until DNA damage is repaired. If the cell cycle is blocked in the G1 or S phase, the damaged bases cannot be copied, thus preventing mutation of the genome and rearrangement of the chromosome structure. When the cell cycle is halted at the G2 stage, double-strand DNA breaks can be made prior to the cell entering mitosis (Fig. 4). The following three tumor suppressor proteins play key roles in detecting DNA damage: ATM/ATR, ChK1/2, and p53 [96–98]. When DNA is damaged, the DNA damage checkpoint is triggered, leading to the activation of the Chk2 protein kinase. This activation results in the phosphorylation and subsequent degradation of the Cdc25phosphatase through polyubiquitination. The inactivation of Cdc25 causes CDK2 to become inactive, halting the cyclin E/A-CDK2-driven transition through the G1 or S phase, thus arresting the cell in these phases [99]. Activated ATM/ATR can also stabilize the p53 protein, which is otherwise highly unstable in the cell, by phosphorylation, resulting in an increased ability to promote the transcription of certain specific genes [100]. DNA damage also activates ATM and CHK2, impacting the stability of node‒microtubule attachments via Aurora A and PLK, leading to increased late lagging chromosomes and chromosome segregation errors during mitosis, which are common in some tumor cells [101]. CHK1 directly phosphorylates and activates WEE1, which in turn increases the inhibitory phosphorylation of CDK2 and CDK1 at Tyr15, causing cell cycle arrest in the G2 phase [102]. It is difficult to determine whether there is a link among CHK1, WEE1, and tumorigenesis, although reduced CHK1 levels (caused by the heterozygous deletion of CHK1) may promote tumorigenesis [103–105]. Unlike CHK1, CHK2 is considered primarily a tumor suppressor. WEE1 expression varies across different tumors but is generally regarded as an oncogene and a potential therapeutic target for cancer [106–109]. Recently, old astrocyte specifically induced substance (OASIS) has been identified as a new therapeutic target for cancer treatment. The DNA damage response (DDR) occurs following the arrest of the cell cycle in the G2/M phase and is controlled by OASIS or p53 [110]. P53 is also involved in tumor cell cycle regulation through the ferroptosis pathway [111]. Recently, several new targets involved in DNA repair reactions have been discovered. Synthetic lethality is an early identified therapeutic mechanism for oncology drugs; however, owing to the lack of suitable targets and tools, this approach has only been studied and used in recent years. PARP (poly ADP-ribose polymerase) is an important enzyme in the DNA repair process, and the recent applications of PARP inhibitors in tumor therapy have been previously reviewed, providing new ideas for oncology drug development [112].

The cell cycle checkpoints and their functions are summarized in Table 2.

**Table 2:** Cell cycle checkpoints and their functions

Checkpoint	Role and features	Main proteins
Unreplicated DNA checkpoint	Monitoring DNA replication and determining whether a cell enters M phase	ATR: Responds to DNA replication stress and DNA damage
		CHK1: Required for checkpoint-mediated cell cycle arrest in response to DNA damage or the presence of unreplicated DNA
		Cyclin A/B-CDK1: Activated CDK1 (cdc2), which binds to cyclin B, drives cells into mitosis...
Spindle assembly checkpoint	Monitoring spindle assembly and determining whether a cell enters late cytokinesis	Mad2: During mitosis, unattached filaments trigger the spindle assembly checkpoint by promoting the assembly of the mitotic checkpoint complex, a heterotetramer containing Mad2, Cdc20, BubR1 and Bub3
		APC: The spindle assembly checkpoint is an essential activator of the ubiquitin ligase late splitting facilitation complex or cyclosome (APC/C)...
Chromosome segregation checkpoint	Monitoring the position of chromosomes in the cell of the zygote at the end of prophase and determining whether the cell progresses to the end of cytokinesis and undergoes cytoplasmic division	Cdc14: Inactivates CDK and controls the timing of mitosis
		Cyclin of M phase...
DNA damage checkpoint	Monitoring the repair of DNA damage and deciding whether the cell cycle continues or stops	ATM/ATR: Plays a crucial role in maintaining genome stability
		Chk1/2: Signal the initiation of the repair process
		p53: Activation of p53 regulates CDK activity, thereby inhibiting cell cycle progression
		Cdc25: Activates the cell cycle protein B1/CDK1 complex in cells to enter mitosis and regulates G2/M progression
		Cyclin E-CDK2: The Cyclin E/CDK2 complex controls cell cycle progression and DNA replication mainly through phosphorylation of specific substrates...

## Conclusions and Perspectives

Although cell cycle regulators were identified years ago, their significance in cancer and potential as therapeutic targets have only recently been revealed. Because current research is inadequate and the treatment of malignant tumors is a challenge, it will therefore take a long time to complete this work. The present review provides an overview of the cell cycle and the link between cell cycle-related proteins and tumors. The present article also provides a brief overview of some oncology drug therapeutic targets and current advances. Interestingly, a previous has indicated that there is also a checkpoint in the G1 phase that effectively prevents mitotic defects [124]. These findings suggest that there are many more potential therapeutic targets for cell cycle-related proteins (cyclins, CDKs, and CDK inhibitors) in the treatment of malignant tumors. These findings suggest that there are still many unknowns related to the cell cycle remain to be defined. The advancement of cell cycle-focused treatments will hinge on creating highly selective medications and pinpointing weaknesses in cancer cells. Numerous clinical studies are currently in progress; however, additional evidence-based research will be essential in the future to confirm the precision of specific targets. More research is needed to address how to ensure the effectiveness and safety of drugs and resistance to subsequent treatments.

Future research efforts should focus on clarifying the pathogenesis of malignant tumors and developing more targeted drugs for novel therapeutic regimens. These findings provide new hope for the treatment of tumors in the future.

## Data Availability

Data sharing is not applicable to this article, as no datasets were generated or analyzed during the current study.
